# Acute Immune Cell Dynamics During Myocardial Infarction and Their Association with Mortality

**DOI:** 10.3390/ijms26136543

**Published:** 2025-07-07

**Authors:** Harris Avgousti, Reina Nagasaka, Adovich S. Rivera, Anna Pawlowski, Edward B. Thorp, Matthew J. Feinstein

**Affiliations:** 1 Feinberg School of Medicine, Northwestern University, Chicago, IL 60611, USA; harris.avgousti@northwestern.edu; 2 Division of Cardiology, Department of Medicine, Bluhm Cardiovascular Institute, Northwestern University Feinberg School of Medicine, Chicago, IL 60611, USA; reina.nagasaka@my.rfums.org; 3 Department of Medical Social Sciences, Feinberg School of Medicine, Northwestern University, Chicago, IL 60611, USA; adovich.rivera@northwestern.edu; 4 Northwestern Medicine Enterprise Data Warehouse, Chicago, IL 60611, USA; apawlows@nm.org; 5 Department of Pathology, Northwestern University, Chicago, IL 60611, USA; ebthorp@northwestern.edu; 6 Division of Cardiology, Department of Medicine, Northwestern University, Chicago, IL 60611, USA

**Keywords:** myocardial infarction, neutrophils, immune cells, inflammation, troponin

## Abstract

Acute neutrophil responses following myocardial infarction (MI) play a central role in remodeling, contributing to both repair and potential maladaptive responses. Although prior studies have investigated circulating immune cell indices at a single time point during hospitalization for MI, limited data exist on acute intra-individual changes in circulating immune profiles during evolving MI. We analyzed clinical measurements, such as the count and proportion of immune cell components in a serial complete blood count, with differential tests conducted for patients hospitalized with ST-elevation MI (STEMI) in various hospitals in the Northwestern Medicine system from 1 January 2002 to 1 August 2024. Patients with STEMI diagnosis, troponin peaks ≥ 5 ng/mL, and cell count and proportion data prior to the troponin peak and within 24 h after the troponin peak were included. Primary analyses investigated the associations between the troponin peak and peri-STEMI changes in immune cell subsets. Multivariable-adjusted Cox models were used to investigate associations between these peri-STEMI immune cell changes and mortality at 1 year and 3 years. Among the 694 STEMI patients meeting the inclusion criteria, a higher troponin peak was associated with a modest peri-MI increase in neutrophil proportion. Higher adjusted peri-STEMI increases in neutrophil count and proportion were strongly associated with mortality at one and three years [hazard ratio (HR) = 1.31 (95% confidence interval (CI) 1.15–1.49) and HR = 1.27 (95% CI 1.14–1.45) per 1000 cells/μL absolute neutrophil increase, respectively]. Individuals with higher STEMI-related neutrophil increases had higher mortality at one year and three years, independent of the extent of troponin elevation.

## 1. Introduction

Systemic and myocardial inflammation plays a central role in the pathophysiology of myocardial infarction (MI) and post-MI recovery. Following the ischemic insult of MI, there is a rapid and robust activation of the innate immune system, with leukocyte mobilization critical to both injury propagation and subsequent repair. In the 24 to 48 h after MI, acute leukocytosis occurs, characterized by an initial surge in circulating neutrophils followed by a rise in monocytes [1,2,3]. These myeloid-derived cells originate from hematopoietic reservoirs such as the bone marrow and spleen and are actively recruited to the ischemic myocardium via chemokine-mediated signaling [4,5]. While their role is initially reparative—clearing necrotic debris and promoting tissue remodeling—prolonged or dysregulated inflammation can contribute to adverse left ventricular (LV) remodeling, scar expansion, and ultimately, heart failure (HF) [6,7].

Prior observational studies have demonstrated that elevated neutrophil counts or neutrophil-to-lymphocyte ratios (NLRs) at the time of MI are associated with an increased risk of subsequent HF and mortality [8,9]. These findings underscore the potential of neutrophils to act as biomarkers of adverse outcomes. However, these studies generally rely on static measurements of immune cells at a single time point—either pre- or post-MI—which limits our understanding of the dynamic trajectory of leukocyte subsets in the acute peri-MI window. Such temporal patterns may more accurately reflect the inflammatory phenotype of individual patients and its relationship to myocardial injury and recovery [10,11].

Recent studies suggest that immune cell kinetics—especially early shifts in neutrophil and monocyte populations—may influence infarct healing and predict long-term outcomes, such as LV dysfunction and mortality [12]. Yet, few investigations have leveraged serial immune profiling to explore how MI severity and clinical covariates relate to these dynamic changes and whether such shifts are independently associated with post-MI survival [13,14].

In this study of patients with ST-elevation myocardial infarction (STEMI) confirmed by laboratory markers of acute myocardial injury, we leveraged high-resolution, individual-level serial measurements of circulating immune cell subsets. Our goals were twofold: first, examine how MI severity and clinical features influence acute changes in immune subsets, and second, assess the prognostic significance of these immune responses—particularly the peri-STEMI dynamics of neutrophils and monocytes—on post-MI mortality.

### Perspective

We observed that higher intra-individual serial increases in neutrophil counts and proportions within 24 h of ST-elevation myocardial infarction are significantly associated with mortality, independent of the extent of troponin elevation.

These findings suggest that acute changes in circulating immune subsets during myocardial infarction may provide important prognostic information for subsequent adverse events.

Future investigations of the factors driving acute alterations in neutrophil response during myocardial infarction, as well as how intervening in these responses may alter post-myocardial infarction remodeling and outcomes, may be warranted.

## 2. Results

### 2.1. Sample Characteristics

**Figure 1:**
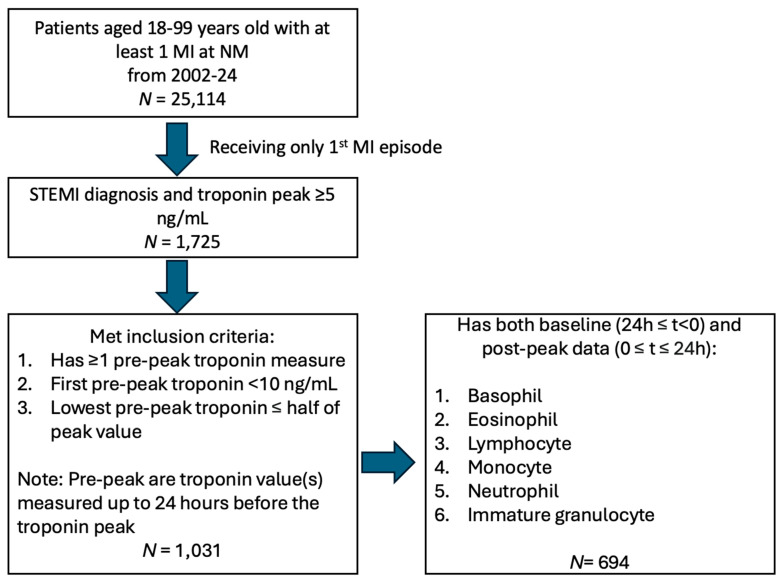
Selection of patients. Between 1 January 2002 and 1 August 2024, 1725 patients were admitted to Northwestern Medicine (NM) for an ST-elevation myocardial infarction (STEMI) with a troponin peak ≥ 5 ng/mL, of whom 694 were included in this study. These patients had a corresponding complete blood count (CBC) with differential tests completed for baseline and post-troponin peaks during the hospital admission.

The study design and cohort selection method is shown in Figure 1. After screening 25,114 individuals with a diagnosis of MI in the NMEDW, all individuals who did not have a STEMI diagnosis and troponin peak of ≥5 ng/mL were excluded, leaving 1725 individuals with STEMI. Of these, 1031 individuals met the additional inclusion criteria of having pre-peak troponin levels measured < 10 ng/mL and a lowest pre-peak troponin level less than or equal to half of the peak troponin value. Finally, when we further restricted analyses to individuals with a complete immune count and proportional data both pre- and post-peak during the STEMI hospitalization, 694 individuals remained and were included for analysis.

Of the 694 STEMI patients across the Northwestern Medicine system included for analysis (Table 1), approximately one-third were hospitalized at Northwestern Memorial Hospital (NMH), an urban tertiary care center, and the other two-thirds were hospitalized at non-NMHs. The cohort had a mean age of 64.57 ± 12.84 years, with non-NMH patients being older (mean 65.64 ± 12.10 vs. 62.32 ± 12.03 years, *p* = 0.001). Demographics differed between the NMH and non-NMH sites, as shown in Table 1. The mean troponin peak of the entire cohort was 52.93 ng/mL, with no significant difference between NMH and non-NMH patients.

Overall, our selection of patients across various centers in the Northwestern Medicine system resulted in 694 patients diagnosed with STEMI, multiple troponin values, and immune cell data available for analysis. Most baseline characteristic data did not significantly differ across patient sites.

### 2.2. Higher Peak Troponin Levels During STEMI Are Associated with a Higher Neutrophil Proportion Increase Within 24 h

We first investigated the associations between peak troponin levels and the post-peak proportions of each immune cell subset, adjusted for the same individual’s baseline proportion of that subset (a “peri-STEMI [cell subset] proportion change or increase”). Each 1 ng/mL higher peak of troponin was associated with a 0.05% peri-STEMI neutrophil proportion increase or a 5% higher neutrophil proportion per 100 ng/mL of the higher troponin peak (*p* < 0.001; Table 2); associations and significance were unchanged with multivariable adjustment. A higher troponin peak was also associated with a significantly higher peri-STEMI immature granulocyte proportion increase (*p* < 0.05; Table 2). Peak troponin levels were not associated with peri-STEMI increases or decreases in other immune cell subsets within 24 h of the troponin peak, including no significant change in monocytes (our other co-primary immune proportion endpoints of interest). Sensitivity analyses that were separately stratified by sex, DM status, BMI group, and medical center (NMH versus non-NMH) yielded similar associations across subgroups (Supplementary Table S2). The troponin peak was not associated with peri-STEMI changes in absolute cell counts for neutrophils or other cell subsets (Table 2).

These data suggest a shift in the relative proportion of peripheral immune cell subsets, including a significant increase in the neutrophil proportion during the peri-STEMI timeframe and a direct association with the increasing troponin peak.

### 2.3. Acute Peri-STEMI Changes in Immune Subsets and Mortality at 1 Year and 3 Years Post-STEMI

**Figure 2:**
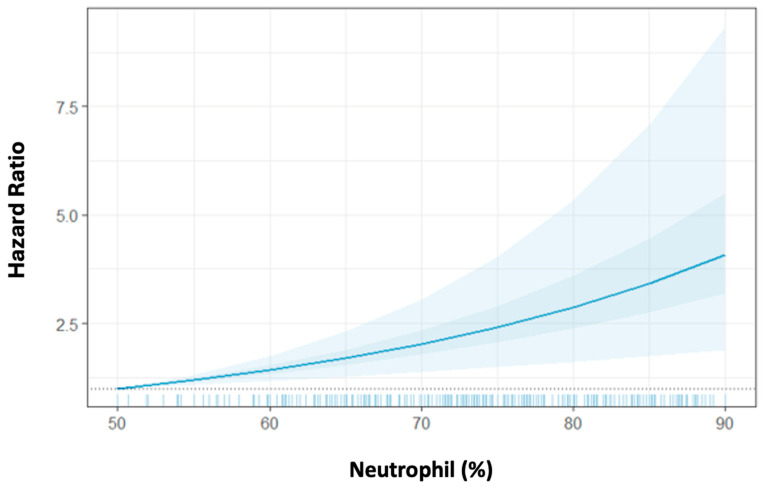
The association between the post-peak neutrophil proportion and the 1-year mortality hazard ratio. Hazard ratios (HRs) for death within 365 days are plotted across post-peak neutrophil proportions ranging from 50 to 90%, using neutrophil (%) = 50 as the reference shown by the dashed line. The light blue shaded area represents the 95% confidence interval (CI) for the HR estimates, illustrating the increase in HR with rising neutrophil (%) levels.

Given the significant association of the troponin peak with the peri-STEMI neutrophil proportion increase, we next assessed the association between post-peak neutrophil proportion with mortality and the extent to which this association is independent of the troponin peak. We observed that a higher peri-STEMI neutrophil proportion increase was associated with significantly increased 1-year mortality after adjustment for age, sex, DM, BMI, the troponin peak, and NLR. The HR for 1-year mortality was 1.05 [95% confidence interval (CI) = 1.03–1.08] per 1% higher post-peak neutrophil proportion, corresponding to a 50% increased risk for mortality per 10% peri-STEMI neutrophil proportion increase (Table 3, Figure 2). Similarly, the higher peri-STEMI neutrophil proportion increase was also associated with a significantly increased 3-year mortality rate after adjusting for covariates. Interestingly, the peri-STEMI neutrophil absolute count increase was also associated with increased 1-year and 3-year mortality rates [HR = 1.31 (95% CI 1.15–1.49) and HR = 1.27 (95% CI 1.14–1.45) per 1000 cells/µL absolute neutrophil increase, respectively]. These neutrophil associations were similar when analyzed separately by each medical center (NMHs vs. non-NMHs; Supplementary Table S3). We also observed that the peri-STEMI absolute monocyte count increased significantly and was associated with 1- and 3-year mortality [HR = 5.18 (95% CI 2.21–12.11) and HR = 4.83 (95% CI 2.14–10.91) per 1000 cells/µL absolute monocyte increase, respectively].

**Figure 3:**
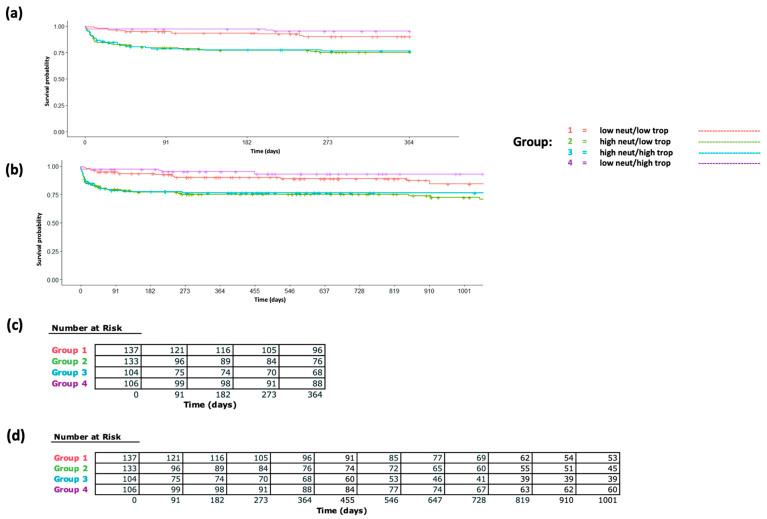
Survival differences between 1 year and 3 years post-STEMI based on post-peak neutrophil proportions and troponin peaks. The survival curve is shown for a STEMI patient based on the selected post-peak neutrophil proportion (above or at the median level vs. below the median) and selected troponin peak (above or at the median level vs. below the median) at (**a**) 1-year post-STEMI and (**b**) 3-years post-STEMI. The figure legend is shown to the right. For each panel, survival curves were derived from the multivariable Cox proportional hazard model for the high vs. low neutrophil group and troponin peak group. A corresponding table is given with the number of patients surviving per group for (**c**) 1-year post-STEMI and (**d**) 3-years post-STEMI. Abbreviations—neut, neutrophil; trop, troponin.

To further investigate the extent to which these acute STEMI-associated immune cell proportions and count changes are associated with mortality independently from the troponin peak, we split our cohort based on median levels of the troponin peak and peri-STEMI neutrophil proportion increase and analyzed survival differences (Figure 3a,b) and mortality hazard ratios (Supplementary Table S4). In multivariable-adjusted Cox proportional hazards models, individuals with a lower (less than median) peri-STEMI neutrophil proportion change and lower (less than median) troponin peak were used as reference, and individuals in the significant neutrophil increase/high troponin peak group, significant neutrophil increase/low troponin peak group, and insignificant neutrophil increase/high troponin peak group had hazard ratios of 2.78 (95% CI 1.44, 5.4), 2.54 (1.27, 5.08), and 0.5 (0.18, 1.41), respectively, for 1-year mortality. Findings were similar for 3-year mortality. Finally, in our separate Cox models exploring the relative predictive value of acute peri-STEMI neutrophil changes for mortality (Supplementary Table S5), the model without this parameter had a C-statistic of 0.62, whereas the model incorporating post-troponin-peak neutrophil proportions had a C-statistic of 0.74, indicating significant improvement in predictive value when incorporating acute peri-STEMI neutrophil changes.

Our results highlight the association between neutrophil (%) and mortality risk in the peri-STEMI time period. We found that higher peri-STEMI neutrophil proportions were significantly associated with increased mortality at 1 and 3 years, independent of the troponin peak.

## 3. Discussion

In this study of patients with STEMI, we observed that acute serial changes in neutrophil proportion and count were significantly associated with mortality at 1 and 3 years. Furthermore, these associations remained consistent and significant after adjusting for common clinical covariates as well as troponin peaks, as demonstrated by secondary analyses treating the troponin peak as a continuous variable for adjustment as well as in the analysis of mortality for individuals grouped by high versus low troponin peaks and high versus low acute peri-STEMI neutrophil changes. Taken together, these findings suggest that STEMI-related changes in circulating neutrophil counts and proportions may represent an important negative prognostic factor, irrespective of other STEMI-related factors such as the troponin peak.

In addition to the neutrophil findings, we observed that increases in monocyte count were associated with significantly higher mortality at 1 year and 3 years. The hazard ratios were comparatively larger than for neutrophil counts, which likely reflects the variable’s normal distribution rather than being indicative of a stronger effect of one cell subset or another. Normal absolute monocyte counts were 200–800 cells/µL, whereas normal absolute neutrophil counts were 2500–7000 cells/µL; thus, an increase of 1000 cells/µL for monocytes is dramatic, representing a several-fold increase from the normal range, whereas a similar increase in neutrophil counts is less dramatic. Because we focused on the acute peri-STEMI change in cell populations within 24 h and focused on neutrophils as the most acute responder, our secondary analysis focused on neutrophils rather than both neutrophils and monocytes. Therefore, future investigation into how changes in both cell populations may impact post-MI recovery, in addition to a robust review of the existing literature in non-human experimental models, is warranted.

In the context of the prior literature, our findings are plausible and add an important new dimension regarding dynamic peri-MI immune responses. Prior studies showing associations between neutrophil counts or proportions and adverse outcomes, such as heart failure and mortality, either assessed neutrophil proportions at a single time point following MI or at hospital admission [8,9,10]. While these findings suggest the adverse role of neutrophils in the peri-MI setting and underscore the plausibility of our findings, the single assessment of immune cell subsets used makes it possible that chronic differences in immune proportions—rather than MI-related changes—underlie the previously observed association. Meanwhile, our assessment of dynamic, acute intra-individual changes in immune subsets provides new and important insights highlighting how acute cellular alterations during the peri-MI period, e.g., an individual’s peri-STEMI neutrophil elevation, could be associated with post-MI outcomes.

This study has several limitations that should be considered. First, we relied on available laboratory values to define a peak troponin measurement, which may not always reflect the true maximum extent of myocardial injury during STEMI. We aimed to address this by restricting our analyses to individuals with an abrupt (at least two-fold) increase in troponin to a high absolute level (≥5 ng/mL), thus enhancing the likelihood that we included only individuals with significant acute myocardial injury during their STEMI from measurements. This likely resulted in the exclusion of individuals with STEMI who did not have a significant troponin elevation captured due to emergent percutaneous coronary intervention upon presentation without subsequent troponin measurement. A separate unavoidable limitation relates to this cohort. Although we included patients from multiple institutions, all were within the Northwestern Medicine health system, which may limit generalizability. Nevertheless, our sensitivity analyses somewhat enhance external validity, as we observed that key associations between the troponin peak and post-peak neutrophil elevations are similar for NMH versus non-NMH sites despite these sites’ systematically differing in patient populations (Supplementary Table S2). Our findings remained consistent and robust across a diverse spectrum of patient demographics and risk factors. Our selection criteria only included STEMI patients while excluding non-ST elevation (NSTEMI) cases, which may introduce selection bias since STEMI is generally associated with greater myocardial damage and higher 28-day mortality [15,16]. Finally, while our immune cell measurements were derived from standardized complete blood counts with a differential performed in clinical laboratories, we did not have the ability to assess additional markers of interest (such as neutrophil activation or migration markers), thus limiting granular insights beyond the immune cell phenotype.

## 4. Materials and Methods

### 4.1. Study Design and Data Source

This retrospective cohort study of prospectively collected data investigating immune cell subset dynamics in the peri-STEMI period study was approved by the Northwestern University Institutional Review Board (IRB# STU00214494). Demographic and clinical data from STEMI patients were extracted through the Northwestern Medicine Enterprise Data Warehouse (NMEDW), which codifies the electronic health records of patients in a searchable format. NMEDW is an integrated database of clinical and research data from patients receiving treatment through Northwestern Medicine (NM) healthcare affiliates and distinct sites throughout the Northeastern Illinois region. These affiliate sites are within metropolitan Chicago, IL, USA: NM Prentice Women’s Hospital, NM Central DuPage Hospital, NM Delnor Hospital, NM Huntley Hospital, NM McHenry Hospital, NM Woodstock Hospital, NM Valley West Hospital, NM Kishwaukee Hospital, Northwestern Memorial Foundation and NM Lake Forest Hospital, Northwestern Memorial Hospital (NMH), and Northwestern University Feinberg School of Medicine.

### 4.2. Study Population

From the NMEDW, we queried patients who were admitted to hospitals within the NM system with STEMI between 1 January 2002 and 1 August 2024. Eligibility criteria included patients aged 18 to <100 years who were not institutionalized in a prison and had non-missing race data. We included the first STEMI hospitalization in the NM health system based on validated administrative codes (see Supplementary Table S1 for a list of ICD-9 and ICD-10 codes relevant for clinical diagnoses) [17].

We further restricted the cohort in several ways to enhance internal validity. To increase the likelihood that STEMI cases were restricted to those with an abrupt rise in troponin consistent with significant, acute myocardial injury, we further restricted the analysis to patients with troponin values during their index STEMI hospitalization that met each of the following criteria: (1) a troponin peak ≥ 5 ng/mL (≥5000 ng/L) during their STEMI hospitalization; (2) at least one additional troponin measurement within 24 h before the peak that was less than or equal to half the troponin peak value; and (3) a first baseline/pre-peak troponin value < 10 ng/mL [18]. For those who met these criteria, to ensure serial immune cell measurement data, we only included STEMI patients who had both a baseline and post-troponin peak complete blood count (CBC) with a differential. For survival analyses of post-MI mortality, we only included individuals who survived at least 24 h after the troponin peak. For individuals with several qualifying STEMI events over distinct hospitalizations, we only included their first such STEMI for this analysis. Distinct admissions that were less than 24 h apart were merged and treated as a single admission.

### 4.3. Immune Cell Components

We extracted white blood cell (WBC) differential count data from NMEDW to obtain the percentages (%) and counts of neutrophils, monocytes, and immature granulocytes as well as neutrophil–lymphocyte ratios (NLR) at the peri-MI period. We identified both the baseline and post-peak values of these immune cell components. The baseline is the last measurement during the 24 h period before the troponin peak. The post-peak value is the last measurement from the time of the troponin peak up to 24 h after the peak.

### 4.4. In-Health System Mortality

We assessed mortality from 24 h post-troponin peaks for up to 1 year and 3 years of follow-up. Death data was based on mortality recorded by the NMEDW for a clinical inpatient or outpatient record of death.

### 4.5. Statistical Analysis

Our first set of analyses assessed the associations of troponin peak values with the changes in immune cell subsets described above, with our pre-specified co-primary endpoints for these analyses being neutrophil and monocyte subsets as a proportion of total WBCs. We used linear regression models that included both the post-peak and mean-centered baseline values of the immune cell component of interest (e.g., neutrophil (%)) as predictors [19]. We also ran the same models but adjusted for sex and age. Separate models were calculated for each immune cell component. Because we were interested in the dynamics of circulating immune cells peri-MI, each post-peak immune cell proportion was normalized to the same individual’s baseline/pre-peak proportion of that immune cell subset; this enabled us to account for baseline differences in WBC subsets and focus on intra-individual changes in WBC subsets during the STEMI period as our primary WBC subset-related variable of interest. Post-peak cell proportions, adjusted for pre-peak proportions for the same individual, are referred to as the “peri-STEMI [cell subset] proportion change or increase” for consistency. Marginal differences were calculated using the marginal effects package [20].

We used Cox proportional hazards models to assess associations between post-peak immune cell proportions and mortality at 1 year and 3 years post-peak with follow-up starting at 1-day post-peak troponin. Records were administratively censored at the earliest date until 12 August 2024 or the date of death. Base models were adjusted for age, sex, diabetes mellitus (DM), and body mass index (BMI), given the potential for these to confound the immune cell proportion–mortality relationship. Primary analyses treated immune cell parameters—count and proportion—as continuous variables, with mortality as the outcome variable. Next, given the expected associations between myocardial injury severity (as proxy-measured by the troponin peak) and peri-MI neutrophil changes, we aimed to disentangle the potential effects of myocardial injury from those of neutrophil changes on mortality in several ways. First, we additionally adjusted the base Cox models for the troponin peak when examining associations between peri-STEMI neutrophil changes and mortality. Separately, we categorized individuals based on the median split of peri-STEMI neutrophil changes and troponin peaks, comparing mortality for each category (a significant neutrophil change and high troponin peak; a significant neutrophil change and low troponin peak; an insignificant neutrophil change and high troponin peak) versus the insignificant neutrophil change and low troponin peak reference group. To further assess if the post-peak neutrophil (%) predicted mortality independent of the troponin peak, we calculated the C-statistics of three Cox models with varying specifications based on the presence or absence of troponin and neutrophil parameters [21]. The first model included a troponin peak and baseline neutrophil (%), the second model was adjusted for the troponin peak, baseline neutrophil (%), and post-peak neutrophil (%), and the third model was adjusted for baseline and post-peak neutrophils (%)—all models were adjusted for legal sex, DM status, and BMI.

Given the fact that multiple hospitals and medical centers contributed data, we also performed sensitivity analyses stratified by patients admitted to the NMH, an urban tertiary care facility in Chicago, IL, versus non-NMH patients; the purpose of this was to ensure that findings were consistent in non-randomly different settings, thus avoiding bias toward optimism if similar findings were observed across distinct settings. Additionally, to test whether associations between neutrophil count or proportion and mortality were consistent across centers (NMH versus non-NMH patients), we employed Cox proportional hazard models incorporating interaction terms between the site and neutrophil metrics while allowing for site-specific baseline hazard functions [22]. The model fit improvement from the inclusion of the interaction term was evaluated using ANOVA tests (using *p* < 0.10 as the threshold). When interactions were significant, site-specific hazard ratios (HRs) were calculated using linear combinations of model coefficients. Survival analyses and related C-statistics were computed using the survival package [23]. HRs from models with interaction terms were calculated using the glht function in the multcomp package [24].

## 5. Conclusions and Clinical Perspective

In an analysis of multiple medical centers within a single health system, we aimed to study how immune cell subsets associate with troponin peak and long-term mortality in the peri-MI timeframe. We observed that STEMI-related neutrophil increases are independently proportional to peak troponin in the acute timeframe—more severe myocardial infarction results were seen in cases with increased acute peripheral neutrophil mobilization. We are also the first to show that the acute neutrophil proportion following peak troponin is significantly associated with mortality at 1 year and 3 years and is so independent of the troponin peak. In the context of the prior literature, this suggests a pathological role behind peripheral neutrophil increases in the setting of MI and warrants further mechanistic and clinical study.

## Figures and Tables

**Table 1:** Baseline characteristics of patient cohort.

	Overall (N = 694)	Non-NMH (N = 469)	NMH (N = 229)	*p*-Value
**Age at MI (years)**	64.57 (12.84)	65.64 (12.1)	62.32 (14.03)	0.001
**Male legal sex, n (%)**	490 (70.6)	332 (70.8)	158 (70.2)	0.949
**Race & ethnicity, n (%)**				<0.001
AAPI/AIAN	41 (6.5)	25 (5.7)	16 (8.6)	
Black	53 (8.5)	14 (3.2)	39 (21.0)	
Hispanic	35 (5.6)	23 (5.2)	12 (6.5)	
White	497 (79.4)	378 (85.9)	119 (64.0)	
**Insurance category, n (%)**				0.058
Commercial	288 (41.7)	184 (39.2)	104 (46.8)	
Medicaid	65 (9.4)	46 (9.8)	19 (8.6)	
Medicare	315 (45.6)	227 (48.4)	88 (39.6)	
Other	0 (0.0)	0 (0.0)	0 (0.0)	
Uninsured	23 (3.3)	12 (2.6)	11 (5.0)	
**Commercial insurance, n (%)**	288 (41.7)	184 (39.2)	104 (46.8)	0.07
**Number of pre-admission visits, n (%)**				
Outpatient	34.80 (55.73)	22.95 (37.34)	56.45 (74.40)	<0.001
Emergency department	2.52 (2.86)	2.48 (2.77)	2.60 (3.09)	0.775
Inpatient	3.83 (5.43)	2.85 (2.91)	5.32 (7.63)	0.001
**BMI (kg/m^2^)**	29.48 (6.06)	30.00 (5.97)	28.40 (6.11)	0.001
**Troponin peak (ng/mL)**	52.93 (56.41)	52.68 (58.20)	53.45 (52.58)	0.865
**Baseline WBC**				
WBC count (10^3^ cells/μL)	11.42 (12.93)	11.12 (4.36)	12.05 (21.94)	0.38
Basophils (%)	0.58 (1.02)	0.68 (1.19)	0.36 (0.43)	<0.001
Eosinophils (%)	1.80 (1.69)	1.92 (1.66)	1.53 (1.74)	0.005
Lymphocytes (%)	25.62 (15.45)	25.34 (13.93)	26.19 (18.21)	0.498
Monocytes (%)	7.11 (3.01)	7.18 (2.80)	6.98 (3.42)	0.402
Neutrophils (%)	64.33 (16.79)	64.34 (15.63)	64.30 (19.07)	0.979
Immature Granulocytes (%)	0.58 (0.78)	0.59 (0.79)	0.55 (0.77)	0.554
Absolute Basophil Count (10^3^ cells/μL)	0.06 (0.14)	0.07 (0.17)	0.03 (0.04)	0.007
Absolute Eosinophil Count (10^3^ cells/μL)	0.19 (0.19)	0.20 (0.19)	0.16 (0.20)	0.067
Absolute Lymphocyte Count (10^3^ cells/μL)	2.60 (2.85)	2.53 (1.49)	2.78 (4.66)	0.336
Absolute Monocyte Count (10^3^ cells/μL)	0.73 (0.36)	0.74 (0.30)	0.70 (0.46)	0.22
Absolute Neutrophil Count (10^3^ cells/μL)	7.09 (3.88)	7.21 (3.77)	6.76 (4.18)	0.241
Absolute Immature Granulocyte Count (10^3^ cells/μL)	0.07 (0.12)	0.07 (0.12)	0.06 (0.12)	0.75

Abbreviations: NMH, Northwestern Memorial Hospital; MI, myocardial infarction; AAPI, Asian American and Pacific Islander; AIAN, American Indian and Alaska Native; BMI, body mass index; WBC, white blood cell count.

**Table 2:** Association between troponin peak value and post-peak WBC subsets.

	Unadjusted	*p*-Value	Adjusted	*p*-Value
**Neutrophil**				
a. Absolute count (10^3^/μL)	0.01 (−0.02, 0.05)	0.404	0.01 (−0.03, 0.05)	0.459
b. Percent (%)	0.05 (0.03, 0.06) *	<0.001	0.05 (0.03, 0.06) *	<0.001
c. NLR	0.02 (0.005, 0.04) *	0.011	0.02 (0.004, 0.04) *	0.012
**Monocyte**				
a. Absolute count (10^3^/μL)	0.001 (−0.025, 0.03)	0.93	−0.00014 (−0.03, 0.03)	0.99
b. Percent (%)	0.0037 (−0.001, 0.01)	0.16	0.0025 (0, 0.01)	0.24
**Immature Granulocyte**				
a. Absolute count (10^3^/μL)	0.000039 (−0.0004, 0.0005)	0.87	0.000061 (−0.0003, 0.001)	0.798
b. Percent (%)	0.001 (0.0001, 0.0019) *	0.024	0.0011 (0.0001, 0.0019) *	0.026

Abbreviations: WBC, white blood cell count; NLR, neutrophil-lymphocyte ratio; * indicates significance at threshold *p* < 0.05; Values represent changes in WBC component per every increase in troponin measurement (ng/mL); Values adjusted for sex, age and mean-centered baseline WBC values.

**Table 3:** Adjusted association of post-troponin peak WBC components with 1- and 3-year mortality.

**1-year mortality**				
	**n**	**events**	**HR**	***p*** **-value**
**Neutrophil**				
Post-peak Count	134	20	1.3 (1.17, 1.46) *	<0.001
Post-peak %	480	74	1.05 (1.03, 1.08) *	<0.001
Post-peak NLR	463	73	1.03 (1.022, 1.05) *	<0.001
Post-peak Count (NLR CV)	134	20	1.31 (1.15, 1.49) *	<0.001
Post-peak % (NLR CV)	463	73	1.03 (0.997, 1.06)	0.075
**Monocyte**				
Post-peak Count	141	20	6.42 (2.89, 14.24) *	<0.001
Post-peak %	486	74	0.89 (0.81, 0.97) *	0.01
Post-peak Count (NLR CV)	138	20	5.18 (2.21, 12.11) *	<0.001
Post-peak % (NLR CV)	465	74	0.92 (0.84, 1.01)	0.09
**Immature granulocyte**				
Post-peak %	358	49	1.3 (0.87, 1.95)	0.205
Post-peak % (NLR CV)	340	49	1.18 (0.78, 1.77)	0.429
**3-year mortality**				
	**n**	**events**	**HR**	***p*** **-value**
**Neutrophil**				
Post-peak Count	134	22	1.27 (1.15, 1.41) *	<0.001
Post-peak %	480	83	1.04 (1.02, 1.07) *	<0.001
Post-peak NLR	463	82	1.03 (1.02, 1.04) *	<0.001
Post-peak Count (NLR CV)	134	22	1.28 (1.135, 1.45) *	<0.001
Post-peak % (NLR CV)	463	82	1.02 (0.99, 1.05)	0.193
**Monocyte**				
Post-peak Count	141	22	5.98 (2.78, 12.89) *	<0.001
Post-peak %	486	83	0.93 (0.85, 1.01)	0.096
Post-peak Count (NLR CV)	138	22	4.83 (2.14, 10.91) *	0.001
Post-peak % (NLR CV)	465	83	0.97 (0.89, 1.06)	0.478
**Immature granulocyte**				
Post-peak %	358	56	1.23 (0.84, 1.81)	0.289

Abbreviations: WBC, white blood cell count; HR, hazard ratio; NLR, neutrophil-lymphocyte ratio; CV, included as covariate; * indicates significance at threshold *p* < 0.05; Cox proportional HR adjusted for age, sex, diabetes status, body mass index, peak troponin.

## Data Availability

The original contributions presented in this study are included in the article/Supplementary Materials. Further inquiries can be directed at the corresponding author.

## Supplementary Materials

The following supporting information can be downloaded at https://www.mdpi.com/article/10.3390/ijms26136543/s1.

## Abbreviations

BMI	body mass index
CI	confidence interval
CBC	complete blood count
DM	diabetes mellitus
HF	heart failure
HR	hazard ratio
LV	left ventricular
MI	myocardial infarction
NLR	neutrophil–lymphocyte ratio
NMH	Northwestern Memorial Hospital
NMEDW	Northwestern Enterprise Data Warehouse
NSTEMI	non-ST elevation MI
STEMI	ST-elevation MI
WBC	white blood cell
