# Supplementary material for: Acute Immune Cell Dynamics During Myocardial Infarction and Their Association with Mortality
Source: Int J Mol Sci. 2025 Jul 7;26(13):6543. doi: 10.3390/ijms26136543 (PMC12249922; doi:10.3390/ijms26136543)
Supplement: Supplementary file 1 [file ijms-26-06543-s001.zip › ijms-3687340-supplementary.pdf]

Supplementary Table S1. ICD codes by diagnosis.

Diagnosis	ICD-9	ICD-10
**STEMI**	41000, 41001, 41002, 41010, 41011, 41012, 41020, 41021, 41022, 41030, 41031, 41032, 41040, 41041, 41042, 41050, 41051, 41052, 41060, 41061, 41062, 41080, 41081, 41082, 41090, 41091, 41092	I2101, I2102, I2109, I2111, I2119, 2121, I2129, I213, I220, I221, I228, I229
**DM**	250, 250.01, 250.01AK, 250.01C, 250.02, 250.03, 250.1, 250.11, 250.12, 250.13, 250.2, 250.21, 250.22, 250.23, 250.3, 250.31, 250.32, 250.33, 250.4, 250.41, 250.42, 250.43, 250.5, 250.51, 250.52, 250.53, 250.6, 250.61, 250.62, 250.63, 250.7, 250.71, 250.72, 250.73, 250.8, 250.81, 250.82, 250.83, 250.9, 250.91, 250.92, 250.93, 250.99	E10.0, E10.10, E10.21, E10.22, E10.29, E10.311, E10.319, E10.321, E10.329, E10.331, E10.339, E10.341, E10.349, E10.351, E10.359, E10.36, E10.39, E10.40, E10.41, E10.42, E10.43, E10.49, E10.51, E10.52, E10.59, E10.610, E10.618, E10.621, E10.649, E10.65, E10.69, E10.7, E10.8, E10.9, E11.00, E11.01, E11.21, E11.22, E11.29, E11.311, E11.319, E11.321, E11.329, E11.331, E11.339, E11.341, E11.349, E11.351, E11.359, E11.36, E11.39, E11.40, E11.41, E11.42, E11.43, E11.44, E11.49, E11.51, E11.52, E11.59, E11.610, E11.618, E11.620, E11.621, E11.622, E11.628, E11.638, E11.641, E11.649, E11.65, E11.69, E11.8, E11.9, E13.00, E13.01, E13.10, E13.11, E13.21, E13.22, E13.29, E13.319, E13.329, E13.339, E13.341, E13.359, E13.39, E13.40, E13.42, E13.43, E13.51, E13.52, E13.59, E13.610, E13.621, E13.628, E13.630, E13.649, E13.65, E13.69, E13.8, E13.9

Abbreviations: STEMI; ST-elevation myocardial infarction; DM, diabetes mellitus.

Supplementary Table S2. Sub-group associations between troponin peak value and post-peak WBC subsets.

**By Sex**								
	**Female**				**Male**			
	**Unadjusted**	***p*-Value**	**Adjusted**	***p*-Value**	**Unadjusted**	***p*-Value**	**Adjusted**	***p*-Value**
**Neutrophil**								
a. Count	0.01 (−0.07, 0.09)	0.754	0.01 (−0.07, 0.09)	0.77	0.01 (−0.03, 0.06)	0.49	0.01 (−0.03, 0.06)	0.494
b. Percent	0.03 (−0.01, 0.06)	0.098	0.03 (−0.01, 0.06)	0.09	0.05 (0.03, 0.07) *	<0.001	0.05 (0.03, 0.07) *	<0.001
c. NLR	0.01 (−0.02, 0.04)	0.658	0.01 (−0.02, 0.04)	0.658	0.02 (0.01, 0.04) *	0.008	0.02 (0.01, 0.04) *	0.008
**Monocyte**								
a. Count	0.00285 (−0.05, 0.06)	0.92	0.0021 (−0.05, 0.06)	0.941	−0.00071 (−0.03, 0.03)	0.962	−0.0007 (−0.03, 0.03)	0.959
b. Percent	0.00007 (−0.01, 0.01)	0.989	0.0003 (−0.01, 0.01)	0.956	0.003 (−0.003, 0.01)	0.312	0.0029 (−0.003, 0.01)	0.333
**Immature Granulocyte**								
a. Count	−0.00012 (−0.001, 0.001)	0.791	−0.000097 (−0.001, 0.001)	0.834	0.000085 (−0.0005, 0.001)	0.763	0.0012 (−0.0003, 0.001)	0.670
b. Percent	0.0016 (−0.0001, 0.003)	0.081	0.0015 (−0.0001, 0.003)	0.070	0.00037 (−0.0002, 0.002)	0.529	0.00035 (−0.0002, 0.002)	0.557
**By DM status**								
	**No DM**				**Has DM**			
	**Unadjusted**	***p*-Value**	**Adjusted**	***p*-Value**	**Unadjusted**	***p*-Value**	**Adjusted**	***p*-Value**
**Neutrophil**								
a. Count	0.02 (−0.03, 0.06)	0.398	0.02 (−0.03, 0.06)	0.458	0.0087 (−0.06, 0.07)	0.801	0.0084 (−0.07, 0.07)	0.809
b. Percent	0.04 (0.02, 0.06) *	0.002	0.04 (0.02, 0.06) *	0.002	0.05 (0.01, 0.09) *	0.032	0.05 (0.01, 0.09) *	0.026
c. NLR	0.03 (0.01, 0.05) *	<0.001	0.03 (0.01, 0.05) *	<0.001	−0.01 (−0.04, 0.02)	0.461	−0.01 (−0.04, 0.02)	0.461
**Monocyte**								
a. Count	0.0012 (−0.02, 0.03)	0.935	−0.00049 (−0.02, 0.03)	0.975	0.0015 (−0.04, 0.04)	0.95	0.0013 (−0.04, 0.04)	0.957
b. Percent	0.0041 (−0.002, 0.01)	0.178	0.0029 (−0.002, 0.01)	0.325	0.000853 (−0.01, 0.01)	0.869	0.00036 (−0.01, 0.01)	0.943
**Immature Granulocyte**								
a. Count	0.00015 (−0.0003, 0.001)	0.619	0.00018 (−0.0002, 0.001)	0.556	−0.00013 (−0.001, 0.0005)	0.703	−0.00014 (−0.001, 0.0005)	0.720
b. Percent	0.0014 (0.001, 0.003) *	0.016	0.00014 (0.001, 0.003) *	0.018	−0.00091 (−0.002, 0.001)	0.347	−0.00093 (−0.002, 0.001)	0.337
**By NMH**								
	**Non-NMH**				**NMH**			
	**Unadjusted**	***p*-Value**	**Adjusted**	***p*-Value**	**Unadjusted**	***p*-Value**	**Adjusted**	***p*-Value**
**Neutrophil**								
a. Count	0.02 (−0.02, 0.06)	0.274	0.02 (−0.02, 0.06)	0.317	−0.00027 (−0.09, 0.08)	0.995	−0.01 (−0.1, 0.08)	0.975
b. Percent	0.05 (0.02, 0.07) *	0.003	0.05 (0.02, 0.07) *	0.002	0.05 (0.02, 0.08) *	0.012	0.05 (0.02, 0.08) *	0.009
c. NLR	0.03 (0.01, 0.05) *	0.005	0.03 (0.01, 0.05) *	0.005	0.01 (−0.02, 0.03)	0.623	0.01 (−0.02, 0.03)	0.623
**Monocyte**								
a. Count	0.0017 (−0.02, 0.03)	0.905	0.00023 (−0.02, 0.03)	0.988	0.0074 (−0.05, 0.05)	0.813	0.0065 (−0.06, 0.05)	0.834
b. Percent	0.0025 (−0.002, 0.01)	0.436	0.0013 (−0.003, 0.01)	0.693	0.0047 (−0.004, 0.01)	0.301	0.0042 (−0.004, 0.01)	0.347
**Immature Granulocyte**								
a. Count	0.00026 (−0.0002, 0.001)	0.333	0.00026 (−0.0002, 0.001)	0.333	−0.00071 (−0.002, 0.0002)	0.159	−0.00065 (−0.002, 0.0003)	0.207
b. Percent	0.00045 (−0.0002, 0.002)	0.462	0.00042 (−0.0003, 0.002)	0.495	0.0012 (−0.0003, 0.003)	0.179	0.0011 (−0.0004, 0.003)	0.187

Abbreviations: WBC, white blood cell count; NLR, neutrophil-lymphocyte ratio; DM, diabetes mellitus; NMH, Northwestern Memorial Hospital; * indicates significance at threshold $p < 0.05$; Values represent changes in WBC component per every increase in troponin measurement (ng/mL); Values adjusted for sex, age and mean-centered baseline WBC values.

Supplementary Table S3. Association of post-peak neutrophil counts and proportions with mortality sub-stratified by hospital site.

**Site-Specifc Hazard Ratios**			***p*-Value for Interaction**
	**Non-NMH**	**NMH**	
**1 Year**			
Count	1.20 (1.13, 1.27, $p < 0.001$)	1.15 (1.07, 1.24, $p = 0.002$)	0.268
Count (NLR CV)	1.17 (1.09, 1.25, $p < 0.001$)	1.13 (1.05, 1.22, $p = 0.002$)	0.355
Percent	1.09 (1.05, 1.13, $p < 0.001$)	1.05 (1.01, 1.08, $p = 0.008$)	0.034 *
Percent (NLR CV)	1.07 (1.03, 1.11, $p < 0.001$)	1.04 (1.004, 1.07, $p = 0.027$)	0.084 *
**3 Year**			
Count	1.18 (1.12, 1.26, $p < 0.001$)	1.13 (1.05, 1.22, $p = 0.001$)	0.217
Count (NLR CV)	1.15 (1.08, 1.23, $p < 0.001$)	1.11 (1.03, 1.19, $p = 0.005$)	0.293
Percent	1.07 (1.04, 1.11, $p < 0.001$)	1.04 (1.01, 1.07, $p = 0.013$)	0.092 *
Percent (NLR CV)	1.05 (1.02, 1.09, $p = 0.003$)	1.03 (1.0001, 1.06, $p = 0.049$)	0.201

Abbreviations: NMH, Northwestern Memorial Hospital; HR, hazard ratio; NLR, neutrophil-lymphocyte ratio; CV, included as covariate; * indicates ANOVA significance at threshold $p < 0.10$; HR represents risk per 1-unit increase in WBC subsets. Absolute counts were measured in cells $\times 10^3/\mu L$, while percent is proportion of total WBC count (%). NLR was measured as the ratio between the absolute neutrophil count to absolute lymphocyte count. Cox proportional HR adjusted for age, sex, diabetes status, BMI, peak troponin.

Supplementary Table S4. Cox proportional hazard ratios of 1- and 3-year mortality based on post-peak neutrophil proportion and troponin peak.

**1-Year Mortality**					
**Neutrophil**	**Troponin Peak**	**Unadjusted HR**	***p*-value**	**Adjusted HR**	***p*-value**
Above/At MD	Above/At MD	2.86 (1.50, 5.44) *	0.001	2.78 (1.44, 5.4) *	0.002
Above/At MD	Below MD	2.73 (1.39, 5.36) *	0.004	2.54 (1.27, 5.08) *	0.008
Below MD	Above/At MD	0.49 (0.17, 1.37)	0.174	0.50 (0.18, 1.41)	0.191
**3-Year Mortality**					
**Neutrophil**	**Troponin Peak**	**Unadjusted HR**	***p*-value**	**Adjusted HR**	***p*-value**
Above/At MD	Above/At MD	2.38 (1.34, 4.26) *	0.003	2.38 (1.34, 4.26) *	0.003
Above/At MD	Below MD	2.08 (1.12, 3.88) *	0.021	2.08 (1.12, 3.88) *	0.021
Below MD	Above/At MD	0.51 (0.21, 1.22)	0.129	0.51 (0.21, 1.22)	0.129

Abbreviations: HR, hazard ratio; MD, median; MD represents median cutoff value; Reference: Below MD neutrophil and Below MD troponin peak; * indicates significance at threshold $p < 0.05$; Post-peak neutrophil (%) was measured as a neutrophil proportion of total WBC count (%), while troponin peak was measured in ng/mL. Each row represents a median-split category of troponin peak and post-peak neutrophil proportion. Cox proportional HR adjusted for age, sex, diabetes status, and BMI.

Supplementary Table S5. Nested cox model concordance analysis of 1- and 3-year mortality prediction based on troponin peak and post-peak neutrophil proportion.

Model Type:	Troponin Peak	Baseline Neutrophil (%)	Post-Peak Neutrophil (%)	1 Year Mortality	3 Year Mortality
Model 1	Yes	Yes	No	0.6162 (0.0328)	0.6014 (0.0320)
Model 2	Yes	Yes	Yes	0.7452 (0.0290)	0.7187 (0.0284)
Model 3	No	Yes	Yes	0.7438 (0.0288)	0.7198 (0.0279)

Neutrophil (%) was measured as a neutrophil proportion of total WBC count (%), while troponin peak was measured in ng/mL. Model 1 adjusts for troponin peak and baseline neutrophil %. Model 2 adjusts for troponin peak, baseline neutrophil %, and post-peak neutrophil %. Model 3 adjusts for baseline neutrophil % and post-peak neutrophil %. All models adjust for sex, age, diabetes status and BMI; values given as a concordance index (standard error of the mean).
